# Angle- and polarization-adaptive aperiodic-anisotropic metasurfaces for broadband reflectance suppression

**DOI:** 10.1016/j.isci.2026.116833

**Published:** 2026-07-17

**Authors:** Jeongbin Yoon, Mingwan Cho, Hyeonhee Kim, Hyeonjin Park, In-Sung Joe, Jonghwa Shin

**Affiliations:** 1 Department of Materials Science and Engineering, Korea Advanced Institute of Science and Technology (KAIST), Daejeon 34141, Republic of Korea; 2 Semiconductor Research Center, Samsung Electronics, Samsungjeonja-ro 1, Hwaseong-si, Gyeonggi-do 18448, Republic of Korea

**Keywords:** aperiodic-anisotropic metasurfaces, broadband antireflection, impedance matching, CMOS image sensors

## Abstract

Optical reflectance at the air-silicon interface degrades CMOS image sensor (CIS) performance, causing signal loss and image artifacts. Conventional single-layer anti-reflective coatings (1-ARCs) offer CMOS-compatible simplicity, but their isotropic nature and limited spatial tunability make them poorly suited to handle angle- and polarization-dependent characteristics of incident light, particularly where the chief-ray angle varies across the sensor surface. Here, we present an aperiodic-anisotropic metasurface (AAM) composed of subwavelength TiO_2_ nanodisks with spatially varying geometric anisotropy, enabling broadband, angle- and polarization-resolved impedance matching tailored to local incidence conditions. Its performance was verified through unit-cell-level optimization and further validated by full-area simulations on a 20 × 20 μm^2^ Si substrate under Gaussian beam illumination, where the AAM achieved ∼1.40% average reflectance across 400–700 nm for both polarizations, outperforming conventional 1-ARC and double-layer anti-reflective coating (2-ARC). This offers a practical solution for CIS and other systems such as LiDAR (light detection and ranging) receivers under spatially varying illumination.

## Highlights

• Aperiodic-anisotropic metasurfaces enable angle/polarization-adaptive antireflection
• Spatially graded TiO_2_ nanodisks achieve broadband impedance matching on silicon
• 1.40% p/s reflectance for Gaussian beam on 20 × 20 μm^2^ silicon at 400–700 nm

## Graphical abstract

**Figure:**
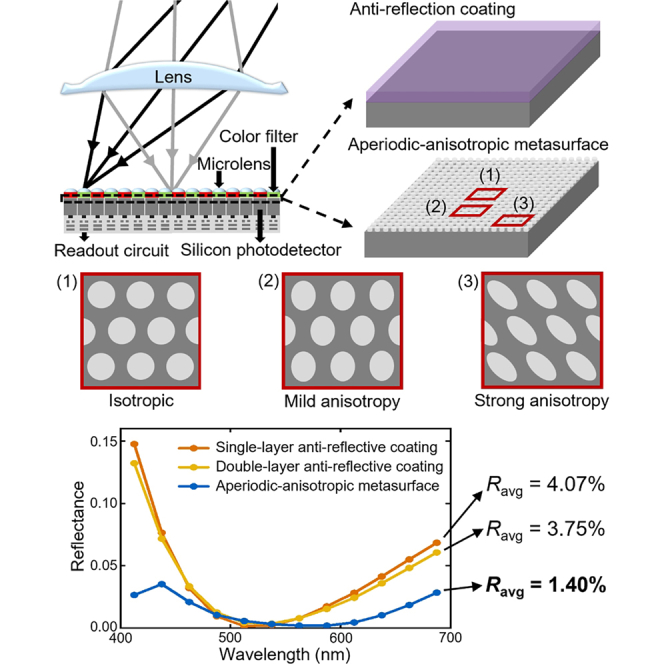

## Teaser

Photonics; Applied sciences; Engineering; Devices

## Introduction

**Figure 1:**
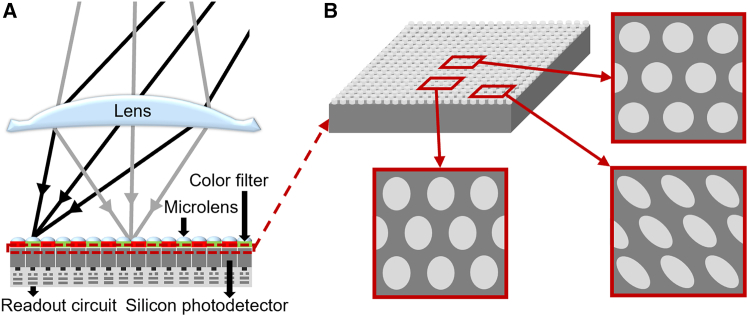
Schematic illustration of the proposed aperiodic-anisotropic metasurface for reflectance minimization in CMOS image sensors (A) Cross-sectional view of light incident on a CMOS image sensor (not drawn to scale), showing position-dependent incidence angles across the photodetector. (B) Top-down view of the AAM, with insets showing the gradual transition from isotropic patterns at the center to increasingly anisotropic patterns toward the edges, adapting to angular variations of incident light.

Complementary metal-oxide-semiconductor (CMOS) image sensors (CISs) are ubiquitous in modern societies, from mobile devices and robotic vacuums to autonomous vehicles and surveillance cameras. A key component of CIS is the silicon (Si)-based photodetector, which, in combination with microlenses and color filters, converts incident light into electrical signals. However, bare silicon exhibits high reflectance (∼40%) in the visible spectrum (400–700 nm) at its interface with air, causing optical losses and introducing optical artifacts such as flare and ghosting that degrade image quality. The reflected and scattered light inside the sensor increases noise, lowering the signal-to-noise ratio (SNR), particularly in low-light or high-contrast conditions. To mitigate reflectance, silicon nitride (Si_3_N_4_) single-layer anti-reflective coatings (1-ARCs) have been widely adopted in CIS fabrication due to their simplicity, CMOS-compatibility, and cost-effectiveness. However, 1-ARCs made of transparent dielectric materials exhibit fundamental limitations. First, although the refractive index of materials such as Si_3_N_4_ can be tuned to some extent by varying gas precursor flows and ratios,^1^^,^^2^ this tuning only adjusts the globally uniform refractive index of the film, and conventional thin-film processes cannot achieve the spatially varying refractive index (*n*) and extinction coefficient (*k*) required for position-dependent anti-reflection. Moreover, its almost non-dispersive optical properties—characterized by a nearly frequency-independent *n* and a very small *k*—leave film thickness as the primary tuning parameter for spectral response, limiting its ability to achieve broadband anti-reflective performance. If this thickness is optimized for minimal reflectance at a certain wavelength, impedance mismatches at shorter and longer wavelengths become larger as the wavelength deviates from the design wavelength, leading to increased reflectance, particularly near both ends of the visible spectrum. Second, the globally uniform thickness of 1-ARC limits its ability to provide optimal anti-reflective performance across the entire surface of a CIS chip. This issue arises because light enters at differing central angles (often called the chief-ray angle) depending on its position on the sensor, as shown in Figure 1A. At the center, light primarily enters at normal incidence, whereas at the edges, oblique incidence dominates. Although a spatially varying 1-ARC thickness could mitigate this issue, it presents significant fabrication challenges. Third, conventional 1-ARC materials are isotropic, meaning they cannot address polarization-dependent impedance matching requirements at oblique incident angles effectively. Even if anisotropic materials were used, there is no readily available fabrication method that allows pixel-wise control of the direction of the crystalline materials’ optical axis, limiting the ability to tailor to angle- and polarization-specific impedance matching conditions. These constraints result in residual reflectance across varying incident angles and polarization states, ultimately reducing CIS performance.

To address some of the shortcomings of 1-ARC, alternative approaches have also been explored, including multilayer coatings,^3^^,^^4^^,^^5^^,^^6^ surface texturing,^7^^,^^8^^,^^9^^,^^10^^,^^11^^,^^12^ nanoparticles,^13^^,^^14^^,^^15^^,^^16^^,^^17^ and more recently, metasurfaces.^18^^,^^19^^,^^20^^,^^21^^,^^22^^,^^23^^,^^24^^,^^25^^,^^26^ While these are promising directions, most prior studies have been designed under the assumption of spatially uniform incident illumination, with structural optimization not tailored to position-dependent incidence. In contrast, the present work addresses the complementary scenario in which the incidence itself varies spatially across the surface—as occurs in imaging sensors, where chief-ray angles vary across the sensor plane. The core innovation of the proposed strategy therefore lies in transitioning from global optimization under uniform illumination to locally adaptive structural design under position-dependent incidence. Importantly, this local adaptation is achieved while maintaining global compatibility in the layer configuration, allowing the entire structure to be fabricated in a single lithography step.

In this study, we propose an aperiodic-anisotropic metasurface (AAM) designed to achieve anti-reflective performance beyond the uniform or isotropic approaches in optical devices on which light is primarily incident in position-dependent angles, such as CIS chips. Unlike conventional coatings and previously explored anti-reflection methods, AAM utilizes chief-ray-optimized subwavelength-scale anisotropic nanostructures to achieve enhanced anti-reflective performance by leveraging their engineered angle- and polarization-dependent impedance characteristics. As shown in Figure 1B, AAM features a gradual transition in nanostructure designs, shifting from isotropic patterns at the center of a sensor chip to increasingly anisotropic structures toward the edges. This structural non-uniformity enables precise tuning of optical properties across the entire surface of the photodetector array, effectively minimizing reflectance over a broad wavelength spectrum and a wide range of incidence angles (up to ±40°) for both p- and s-polarized light. The enhanced anti-reflective performance of the proposed AAM designs was numerically verified using unit-cell-level analysis as well as device-level simulations. Specifically, device-level simulations performed on a 20 × 20 μm^2^ Si substrate under Gaussian beam illumination showed that the AAM achieves an average reflectance of ∼1.40% (averaged over the 400–700 nm range for both polarizations). This significantly outperforms conventional 1-ARC and double-layer anti-reflective coating (2-ARC), which exhibit reflectances of 4.07% and 3.75%, respectively, under the same conditions. With their structurally simple designs, AAM may offer a practical and high-performance anti-reflective solution for CIS and other optical devices, such as silicon-based LiDAR (light detection and ranging) receivers, with stringent requirements on low reflectance.

## Results

### Design and optimization

**Figure 2:**
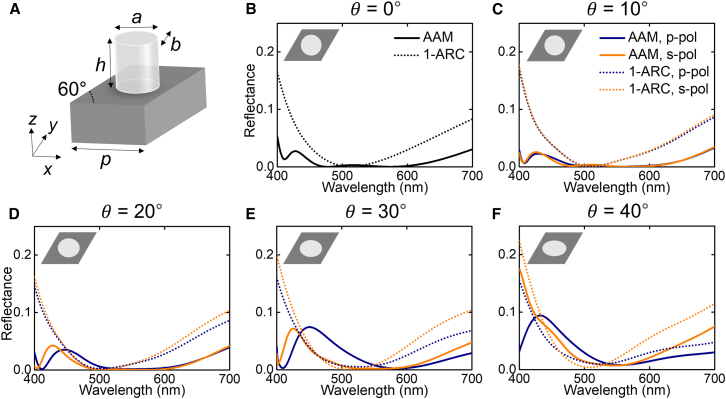
Reflectance spectra of AAM and Si_3_N_4_ single-layer anti-reflective coatings, optimized for various chief-ray polar angles (with zero azimuthal angle, *ϕ*) (A) Schematic of an AAM unit cell, composed of elliptical TiO_2_ nanodisks in a triangular lattice on a silicon substrate. (B–F) Simulated reflectance spectra at chief-ray angles *θ* = 0°, 10°, 20°, 30°, and 40° for both p-polarized (blue) and s-polarized (orange) light. Solid lines correspond to AAM, while dotted lines represent 1-ARC. The inset in each plot shows the optimized nanodisk shape at the corresponding angle, showing the increasing ellipticity with *θ*.

First, we solve a simpler problem of finding an optimized AAM design for a particular, fixed chief-ray angle. The unit cell of the AAM, illustrated in Figure 2A, consists of elliptical TiO_2_ nanodisks arranged in a triangular lattice on a Si substrate. The refractive index data used in the simulations are provided in Figure S1. The AAM structure was defined by four geometric design parameters: the height (*h*), the lengths of the major (*a*) and minor (*b*) axes of the elliptical nanodisk, and the lattice period (*p*). In contrast, the 1-ARC design was characterized by a single tuning parameter—its Si_3_N_4_ film thickness (*t*). Both AAM and 1-ARC structures were optimized using a particle swarm optimization algorithm coupled with finite-difference time-domain (FDTD) simulations. The optimizations were conducted for five polar angles of chief-rays (*θ* = 0°, 10°, 20°, 30°, and 40°) with three azimuthal angles each (*ϕ* = 0°, 15°, and 30°) except for the normal incidence case (*θ* = 0°), resulting in 13 different designs (details described in STAR Methods (unit-cell optimization)). Since we chose a hexagonal lattice, it suffices to check azimuthal angles between 0° and 30° due to symmetries. During optimization, the in-plane rotational angle of the TiO_2_ nanodisk was set equal to the azimuthal angle *ϕ*, aligning the major and minor axes of the nanodisks with the electric field vectors of the incident p- and s-polarized light, respectively. The cost function *F* used in the optimization is defined as Equation 1:

(Equation 1) $$\begin{array}{c} F=\int_{400\;\text{nm}}^{700\;\text{nm}}\left[R_{p}\left(\lambda \right)+R_{s}\left(\lambda \right)\right]\;d\lambda \end{array}$$

where *R*_p_*(λ)* and *R*_s_*(λ)* represent the reflectance for p- and s-polarized light, respectively, at wavelength *λ*. Figures 2B–2F presents the reflectance spectra of the optimized AAM structure for *ϕ* = 0°, evaluated across incidence angles ranging from *θ* = 0° to *θ* = 40°, for both p- and s-polarized light. At normal incidence (*θ* = 0°), for which both AAM and 1-ARC exhibit no polarization dependency, the AAM achieves very low reflectance across the visible spectrum, while the 1-ARC shows relatively high reflectance, especially near the spectral edges. Under oblique incidence (*θ* = 10°, 20°, 30°, and 40°), the AAM consistently exhibits lower reflectance than the 1-ARC for both polarization states. As highlighted in the insets of Figures 2B–2F, the optimized nanodisk shapes become gradually more eccentric as the chief-ray angle increases. This geometric anisotropy, characterized by the gradual increase in the ratio of the nanodisk’s major to minor axes, plays a crucial role in simultaneously minimizing reflectance for both polarizations. A more detailed discussion of how this geometric tuning contributes to reflectance reduction will be explained in the following section. In addition, to assess fabrication tolerance, we examined the effect of geometric variations in nanodisk dimensions for selected optimized AAM designs (*θ* = 0° and 30°), as shown in Figures S2 and S3, which demonstrate robustness against realistic fabrication-induced deviations. The optimized geometric parameters and reflectance values for *ϕ* = 0°, *ϕ* = 15°, and *ϕ* = 30°, along with corresponding data for thickness-optimized Si_3_N_4_ 1-ARC designs, are provided in Table S2.

### Mechanism

**Figure 3:**
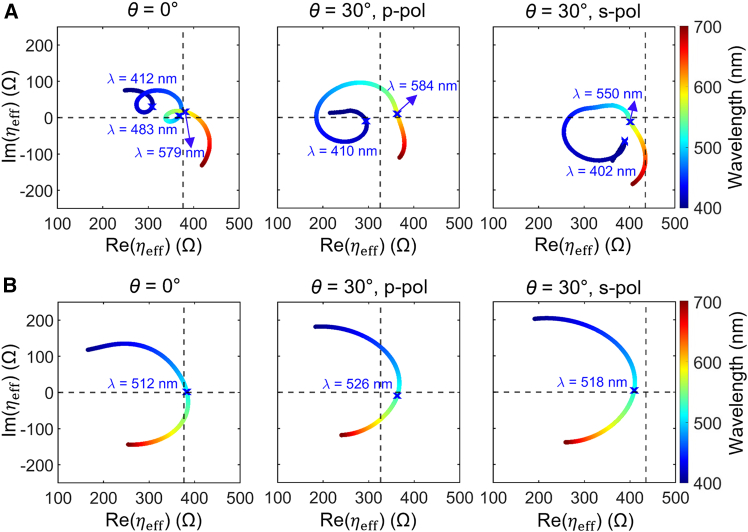
Effective transverse impedance analysis of AAM and 1-ARC Complex-plane trajectories of the effective transverse impedance (*η*_eff_) for (A) AAM and (B) 1-ARC. Both are optimized at *θ* = 0° and *θ* = 30°, on Si substrate. For *θ* = 30°, results are shown separately for p- and s-polarized light. Dashed lines indicate the corresponding transverse impedance of air under each condition. Wavelengths labeled in the plots represent those where local minima in the reflectance appear.

The superior anti-reflective performance of the AAM stems from its ability to adaptively match the optical impedance of air over a broader wavelength range for both polarizations for a given chief-ray angle. To analyze this mechanism, we calculated the effective transverse impedance (*η*_eff_) of the periodic AAM optimized in the previous section, and visualized the results in the complex impedance plane, as shown in Figure 3A. Each trajectory represents a design optimized for either normal (*θ* = 0°) or oblique (*θ* = 30°) incidence; in the latter case, the results were further analyzed for p- and s-polarized light separately. The corresponding transverse impedance values (ratio between the components of electric and magnetic fields that are parallel to the surface) of air under these conditions—376.73 cos(30°) Ω for p-polarized and 376.73/cos(30°) Ω for s-polarized light—are shown as the crossing point of two dashed lines.

The AAM has winding impedance trajectories that stay near the target impedance over a broader wavelength range than that of 1-ARC. This behavior is enabled by multiple resonance modes, supported by the nanostructures. Moreover, for oblique incidences, AAM’s impedance trajectories for two polarizations are clearly displaced from each other on the complex plane, getting closer on average to the corresponding ideal impedance points. In addition, due to the winding nature, each impedance trajectory of AAMs has multiple (three for the normal incidence case and two for *θ* = 30°) “close encounters” with the ideal impedance point as denoted by their wavelengths in the figure, and these wavelengths correlate with the local reflectance dips observed in Figures 2B and 2E. This tuning is made possible by the anisotropic nature of the TiO_2_ nanodisks, which gradually evolve from circular to increasingly elliptical shapes as the incidence angle increases. A detailed analysis of the resonance modes is provided in (Figures S4–S8 and Table S1). The nanodisk array supports a Mie resonance at shorter wavelengths and a Fabry-Pérot (FP) resonance at longer wavelengths, coupled through Fano-type interference. The elliptical geometry introduces anisotropy in the effective index of the array, causing the FP resonance to split between p- and s-polarization, which underlies the independent tuning of the impedance trajectories. The total electric field intensity distributions at representative FP wavelengths (Figure S8) further confirm the polarization-dependent FP resonance conditions. This illustrates the importance of the resonance-mediated impedance matching and anisotropy provided by the elliptical nanodisks, allowing the metasurface to adapt its impedance independently for each incidence angle and polarization—a capability that conventional 1-ARC designs inherently lack.

In contrast, Figure 3B shows the effective transverse impedance trajectories of Si_3_N_4_ 1-ARC designs, each optimized in thickness to minimize the average reflectance at either normal (*θ* = 0°) or oblique (*θ* = 30°) incidence. Its impedance trajectories fail to align with the air impedance, particularly at the shorter and longer wavelengths of the visible spectrum, leading to higher residual reflectance. This limitation arises from the 1-ARC’s lack of material resonances in this frequency range. Figure S9 further confirms this constraint, showing that no single thickness value can perform effectively under all conditions.

These findings demonstrate that the AAM achieves flexible, polarization- and angle-specific impedance matching through structural design. This principle forms the basis for the following section, where we extend this concept into a spatially graded metasurface, tailored to the position-dependent incidence angles present across the photodetector surface.

### Spatially graded metasurface

**Figure 4:**
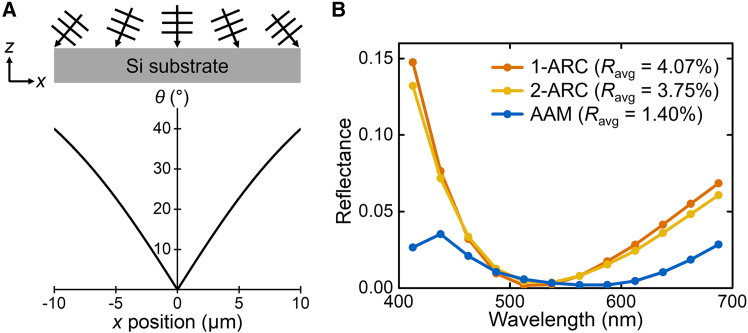
Comparison of reflectance on a Si substrate surface with spatially graded AAM and uniform anti-reflective coatings (A) Schematic of a Gaussian beam illuminating a 20 × 20 μm^2^ silicon substrate, showing a position-dependent incidence angle profile along the *x*-direction at *y* = 0 μm, varying from 0° at the center (*x* = 0 μm) to ±40° at the edges (*x* = ±10 μm). (B) Simulated reflectance spectra (averaged over p- and s-polarizations) across the 400–700 nm range for a spatially graded AAM and uniform 1-ARC and double-layer anti-reflective coating (2-ARC) applied over the same area.

Since the chief-ray angles on a typical CIS chip vary gradually over the surface with a small spatial gradient (<0.05°/μm), it is expected that the optimized nanostructure designs would also vary slowly as a function of position. Thus, AAM designed with locally periodic approximation would perform well on actual CIS chips since neighboring nanodisks would be nearly identical to one another. To verify this and guide the design of a spatially graded AAM, we modeled a 20 × 20 μm^2^ Si substrate surface illuminated by a Gaussian beam. While this model does not fully capture the realistic angular distribution—i.e., a spread of rays centered around the chief-angle ray at each position—it allows us to evaluate the reflectance response under a continuous variation of incidence angles across the device. Moreover, this setup possesses much larger gradient (∼4°/μm) of chief-ray angles than typical CIS chips and, hence, provides a worst-case test bed for locally periodic-approximation-based spatially graded designs. The incidence angle (*θ*) varies along the *x*-direction from 0° at the center (*x* = 0 μm) to ±40° at the edges (*x* = ±10 μm) for *y* = 0 μm, as illustrated in Figure 4A. Additional simulation details are provided in the STAR Methods (full-area device-level simulation).

For a comprehensive device-level performance comparison, three surface configurations on top of the Si substrate were considered: spatially graded AAM, a conventional 1-ARC, and a 2-ARC as a more advanced baseline. In the AAM designs, a fixed nanodisk height (*h* = 91 nm) and lattice period (*p* = 319 nm) were used, considering realistic fabrication constraints where the unit cell pitch and nanostructure height are typically unified. These values correspond to the average of optimal parameters obtained from unit cell simulations at five polar angles (*θ* = 0°, 10°, 20°, 30°, and 40°) and three azimuthal angles (*ϕ* = 0°, 15°, 30°). At this fixed period and height, the nanodisk lateral dimensions—major axis (*a*) and minor axis (*b*)—were re-optimized for each angle combination using the same cost function defined in Equation 1. Based on these discrete design points, the nanostructure geometry was spatially interpolated and extrapolated to generate a smoothly varying metasurface across the Si substrate surface. Rotational symmetry was applied to extend the pattern across azimuthal directions beyond the original simulation set (*ϕ* = 0°, 15°, and 30°), enabling full angular coverage of the surface.

In a practical device, thin-film coatings must maintain a uniform, fixed thickness across the entire substrate. For a fair performance comparison, both the 1-ARC and 2-ARC configurations were optimized using an identical angle-averaged optimization scheme. Specifically, fixed thicknesses—one for the 1-ARC and two for the 2-ARC configuration—were determined by minimizing the reflectance averaged over both polarizations, the 400–700 nm wavelength range, and all incidence angles. These resulting optimal thicknesses were then applied uniformly across the entire surface.

The optimized geometric parameters and corresponding reflectance values for all configurations are summarized in Table S3. We evaluated the reflectance response under Gaussian beam illumination for each configuration—AAM, 1-ARC, and 2-ARC. The resulting spectra in Figure 4B show that the spatially graded AAM achieves a total average reflectance of ∼1.40%, averaged over p- and s-polarizations, the 400–700 nm range, and the 20 × 20 μm^2^ device area, which is notably lower than the 1-ARC (4.07%) and 2-ARC (3.75%). This reflects the AAM’s enhanced anti-reflective performance under spatially varying incidence angles and polarization states, enabled by its ability to locally adapt the nanostructure geometry to both the angular and polarization characteristics of the incoming light.

While Figure 4B confirms the AAM’s effectiveness under spatially varying incidence conditions, real-world illumination often involves a finite angular distribution rather than a single chief-ray. To assess the robustness of the AAM under such conditions, we analyzed the reflectance of a structure corresponding to (*θ* = 27.6°, *ϕ* = 27°), obtained via interpolation from the discrete set of optimized designs used in the spatially graded AAM, under ±10° variations in both polar and azimuthal angles. As summarized in Table S4, the AAM exhibited consistently low average reflectance across the 400–700 nm range for both p- and s-polarizations under these angular deviations, demonstrating strong tolerance to incident light with realistic angular spread. This result reinforces the practical applicability of the AAM design in real optical systems. It is worth noting that while the detailed chief-ray angle distribution depends on the lens curvature and lens-sensor configuration, the proposed design framework does not rely on a specific angular variation profile. Since the AAM is constructed based on a unit-cell database covering a wide angular range (*θ* = 0°–40°), the appropriate nanostructure geometry can be locally selected according to the actual chief-ray angles.

Building on this, the spatially graded metasurface approach demonstrated here is not only compatible with practical CIS, but is also expected to be even more effective when applied to larger-area sensors. As the structural variation becomes more gradual over millimeter- or centimeter-scale sensors, the AAM effectively behaves as a locally periodic structure, further enhancing broadband and angle-resolved impedance matching across the entire device.

## Discussion

This work presents a CMOS-compatible, structurally simple metasurface design that enhances anti-reflective performance under the realistic incidence conditions found in CIS. By employing anisotropic TiO_2_ nanodisks with geometry tailored to local chief-ray angles and polarization states, the proposed AAM enables position-specific impedance matching across the sensor surface. Whereas prior anti-reflection metasurface studies have generally been designed for spatially uniform incident illumination, our spatially graded approach directly addresses the position-dependent chief-ray angles that arise across imaging sensor surfaces, enabling locally adaptive anti-reflection tailored to the incidence conditions at each position. Crucially, this local adaptation is realized while maintaining global compatibility in the layer configuration—a fixed material, uniform nanodisk height, and uniform lattice period, with only the in-plane geometry varying spatially—so that the entire AAM can be fabricated in a single lithography step. Unit-cell simulations verified reflectance suppression across diverse angles and polarizations, and full-area simulations under Gaussian beam illumination further confirmed the effectiveness of a spatially graded AAM. Compared to conventional 1-ARC and 2-ARC designs, the AAM demonstrates notably lower average reflectance across the visible spectrum.

From a fabrication perspective, transitioning from simple thin-film deposition to subwavelength nanodisk arrays inherently entails higher initial manufacturing costs and process complexity, as the small feature sizes require high-resolution patterning techniques such as electron-beam lithography. However, the AAM’s single-layer, unified-height design is specifically tailored to mitigate these challenges. It can be realized by combining standard CMOS-compatible material deposition with scalable, high-throughput patterning techniques such as nanoimprint lithography or deep-ultraviolet steppers. By leveraging these established mass-production routes, the AAM can substantially reduce the per-unit cost penalty. In fact, metasurface-based optical elements have already been incorporated into commercially available image sensors in some consumer mobile devices, indicating that the added fabrication cost is acceptable at industrial scale when the performance benefit is sufficient.

These findings establish AAM as a promising anti-reflective strategy for CIS and other optical systems, such as silicon-based LiDAR receivers, that require robust, polarization-insensitive low-reflectance performance under varying-angle illumination. More broadly, metasurface-based impedance matching has also been explored at other wave interfaces with even stronger impedance mismatch than in the optical case, such as in ultrasound imaging and noise and vibration isolation, to which the impedance-matching principle underlying AAM could likewise be extended.

### Limitations of the study

While this work demonstrates the superior anti-reflective performance of AAM through rigorous numerical analysis, several limitations remain to be addressed. First, this study is primarily based on computational simulations. Although we incorporated realistic material dispersions and performed fabrication tolerance analyses, experimental validation—for example, via electron-beam or nanoimprint lithography—is necessary to confirm the feasibility and scalability of the AAM designs. Second, our device-level simulations utilized a simplified silicon substrate. However, actual CIS often exhibit complex architectures, such as deep trench isolation and intricate readout circuitry, which may introduce additional scattering or parasitic reflections not captured in our flat-substrate model. In practical application, these sub-wavelength features could be treated as an effective medium and incorporated into the optimization to refine the AAM design for such complex CIS architectures. Lastly, the current design assumes air as the background medium above the nanodisk array. In practice, the optical response will be modified by overlying layers such as the encapsulation medium and color filters, requiring further fine-tuning of the nanostructure geometry to match the target system’s integration environment.

## Resource availability

### Lead contact

Further information and requests for resources should be directed to and will be fulfilled by the lead contact, Jonghwa Shin (qubit@kaist.ac.kr).

### Materials availability

This study did not generate new unique reagents.

### Data and code availability

• All data reported in this paper will be shared by the lead contact upon request.
• This paper does not report original code.
• Any additional information required to re-analyze the data reported in this paper is available from the lead contact upon request.

## Acknowledgments

This work was supported by 10.13039/100004358Samsung Electronics Co., Ltd. (IO260312-15859-01). This work was also supported by the 10.13039/501100003725National Research Foundation of Korea (NRF) grant funded by the 10.13039/501100014188Ministry of Science and ICT (RS-2025-02217649) and by the 10.13039/501100003662Korea Evaluation Institute of Industrial Technology (KEIT) grant funded by the Ministry of Trade, Industry, and Resources (RS-2024-00467233, 2410016078).

## Author contributions

J.Y., investigation, formal analysis, software, writing – original draft; M.C., investigation, formal analysis, data curation, supervision, funding acquisition, writing – original draft, writing – review and editing; H.K., software; H.P., methodology; I.-S.J., resources; J.S., conceptualization, supervision, project administration, funding acquisition, writing – original draft, writing – review and editing.

## Declaration of interests

The technology described in this manuscript is covered by a pending Korean patent application (KR 10-2025-0208418). The authors declare no other competing interests.

## Declaration of generative AI and AI-assisted technologies in the writing process

During the preparation of this work, the authors used ChatGPT (OpenAI) and Claude (Anthropic) to assist in writing and debugging parts of the simulation code and for language editing. After using these tools, the authors reviewed and edited the content as needed and take full responsibility for the content of the published article.

## STAR★Methods

### Key resources table

**Table:** 

REAGENT or RESOURCE	SOURCE	IDENTIFIER
**Chemicals, peptides, and recombinant proteins**		

Refractive index data of Anatase TiO_2_	Jolivet et al.^27^	N/A
Refractive index data of Si_3_N_4_	Philipp^28^	N/A
Refractive index data of Crystalline Si	Palik^29^	N/A

**Software and algorithms**		

Ansys Lumerical FDTD	Ansys, Inc.	https://www.ansys.com/
MATLAB R2019b	MathWorks	https://www.mathworks.com/
PSO algorithm script	Robinson and Rahmat-Samii^30^	N/A

### Experimental model and study participant details

No experimental model exists for this work as the study is entirely based on numerical simulations and computational modeling.

### Method details

#### Numerical simulation parameters

Numerical simulations were conducted using a commercial FDTD solver in ANSYS, where periodic boundary conditions were applied along the *x*- and *y*-directions and perfectly matched layer boundary conditions were used along the *z*-direction. The refractive index data for anatase TiO_2_, Si_3_N_4_, and crystalline Si were obtained from established literature and integrated into the simulation environment (see Figure S1 for details).

#### Unit-cell optimization

In unit-cell-level optimization, AAM, 1-ARC, and 2-ARC designs were optimized for fixed chief-ray angles. Linearly polarized plane waves served as the incident source, with polar angles varied in 10° increments (0°–40°) and azimuthal angles *ϕ* set to 0°, 15°, and 30°. The optimization was conducted using a script based on a standard PSO algorithm with absorbing boundary conditions,^30^ executed within the simulation environment. This script was applied independently for each incidence condition to minimize reflectance across the visible spectrum (400–700 nm) for both p- and s-polarized light by tuning the structural parameters. The swarm consisted of 20 particles, and the optimization process was carried out over 50 iterations. To balance global exploration and local exploitation, a linearly decreasing inertia weight was employed, starting at 0.9 and reducing to 0.4 by the final iteration. Both the cognitive and social learning factors were set to 1.49. To ensure broad applicability while preventing unphysical overlapping geometries, a generalized parameter search space was defined for the AAM. The search bounds for the unit-cell period (*p*) and nanodisk height (*h*) were set to 250–400 nm and 50–150 nm, respectively. The lateral dimensions of the elliptical nanodisks, defined by the major axis (*a*) and minor axis (*b*), were parameterized to scale with the unit-cell period using optimization ratios *r*_1_ in [0.25, 0.5] and *r*_2_ in [0.5, 1.0]. Specifically, the axes were determined by the relations *a* = 2*r*_1_*p* and *b* = 2*r*_1_*pr*_2_, which mathematically constrains the nanostructure strictly within the unit cell boundaries. For the 1-ARC and 2-ARC designs, the thickness of each coating layer was optimized within a search bound of 0–100 nm. Post-processing of the optimization results, including the calculation and visualization of the complex-plane impedance trajectories shown in Figures 3 and S9, and the total electric field intensity distributions presented in Figure S8, was performed using MATLAB.

#### Full-area device-level simulation

A large-area (20 × 20 μm^2^) Si substrate was designed, with its surface covered by spatially graded AAM, 1-ARC, and 2-ARC, respectively. A Gaussian beam was launched from *z* = 2 μm toward the Si substrate located at *z* = 0 μm, where the beam width was adjusted such that *W*(*z* = 0) = 6 μm, defined at the 1/e^2^ beam intensity relative to the on-axis peak value. This beam was incident on the entire substrate, generating a spatially varying incidence angle distribution across the surface. By setting appropriate thin lens options in FDTD, we maintained the desired beam divergence within each 25 nm spectral window across the visible spectrum, as the divergence of a Gaussian beam is wavelength-dependent. The reflectance at each spectral point in Figure 4B represents the spectral, spatial, and polarization average over the corresponding 25 nm band. The overall average reflectance values reported in Figure 4B (e.g., *R*_avg_ = 1.40%) are computed by averaging these spectral points across the 400–700 nm range. For clarity, the angular variation of the Gaussian beam at *z* = 0 μm is illustrated in Figure 4A along the *x*-direction at *y* = 0 μm, where the incidence angle ranges from normal incidence (0°) at the center (*x* = 0 μm) to 40° at the edges (*x* = ±10 μm). The spatial interpolation of the optimized nanostructure geometry and post-analysis of the reflectance data were performed within the solver’s analysis framework.

### Quantification and statistical analysis

The anti-reflective performance was quantified by calculating the average reflectance (*R*_avg_) over the visible wavelength spectrum (400–700 nm) for both p- and s-polarizations. All numerical data, including unit-cell optimization results and device-level reflectance for the 20 × 20 μm^2^ area, were processed using the built-in analysis framework of the FDTD solver and MATLAB for further numerical analysis. No additional statistical tests were performed as the data were generated from deterministic numerical simulations.
