# Supplementary material for: Angle- and polarization-adaptive aperiodic-anisotropic metasurfaces for broadband reflectance suppression
Source: iScience. 2026 Jul 17;29(8):116833. doi: 10.1016/j.isci.2026.116833 (PMC13400952; doi:10.1016/j.isci.2026.116833)
Supplement: Document S1. Figures S1–S9 and Tables S1–S4, and Data S1 [file mmc1.pdf]

iScience, Volume *29*

Supplemental information

Angle- and polarization-adaptive

aperiodic-anisotropic metasurfaces

for broadband reflectance suppression

Jeongbin Yoon, Mingwan Cho, Hyeonhee Kim, Hyeonjin Park, In-Sung Joe, and Jonghwa Shin

Supplemental Information

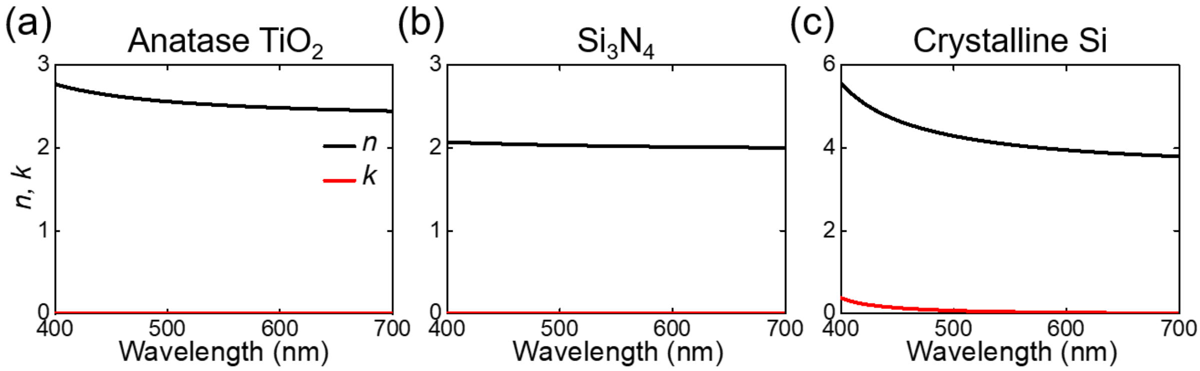

Figure S1. Refractive indices of materials, related to Figure 2.

(a) Anatase TiO_2, (b) Si_3N_4, and (c) crystalline Si. The refractive indices were taken from references [S1]-[S3]. The refractive index of SiO_2 was assumed to be 1.45 without dispersion.

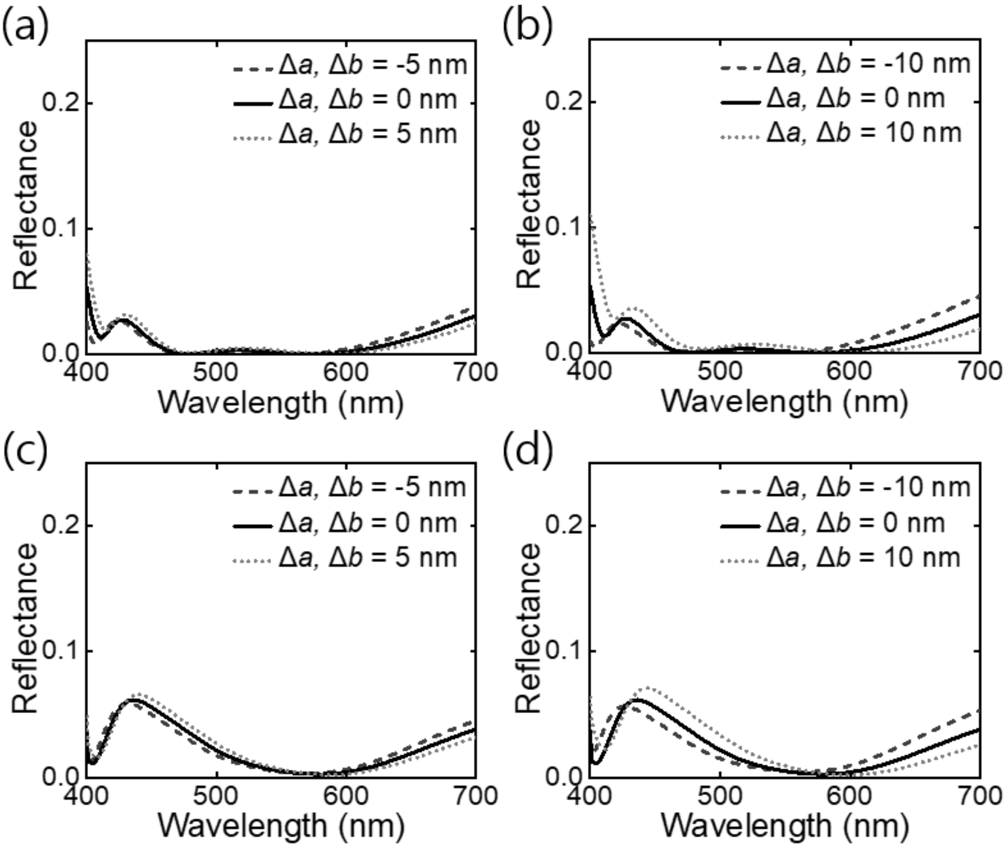

Figure S2. Effect of lateral dimension variations on reflectance, related to Figure 2.

Fabrication tolerance analysis of optimized aperiodic-anisotropic metasurface (AAM) designs at ϕ = 0° for θ = 0° (panels (a), (b)) and θ = 30° (panels (c), (d)). Starting from the optimized dimensions (solid lines), the major and minor axis lengths were simultaneously perturbed by ±5 nm (panels (a), (c)) and ±10 nm (panels (b), (d)), while all other parameters were kept

unchanged. Dotted lines represent increased dimensions and dashed lines represent decreased dimensions.

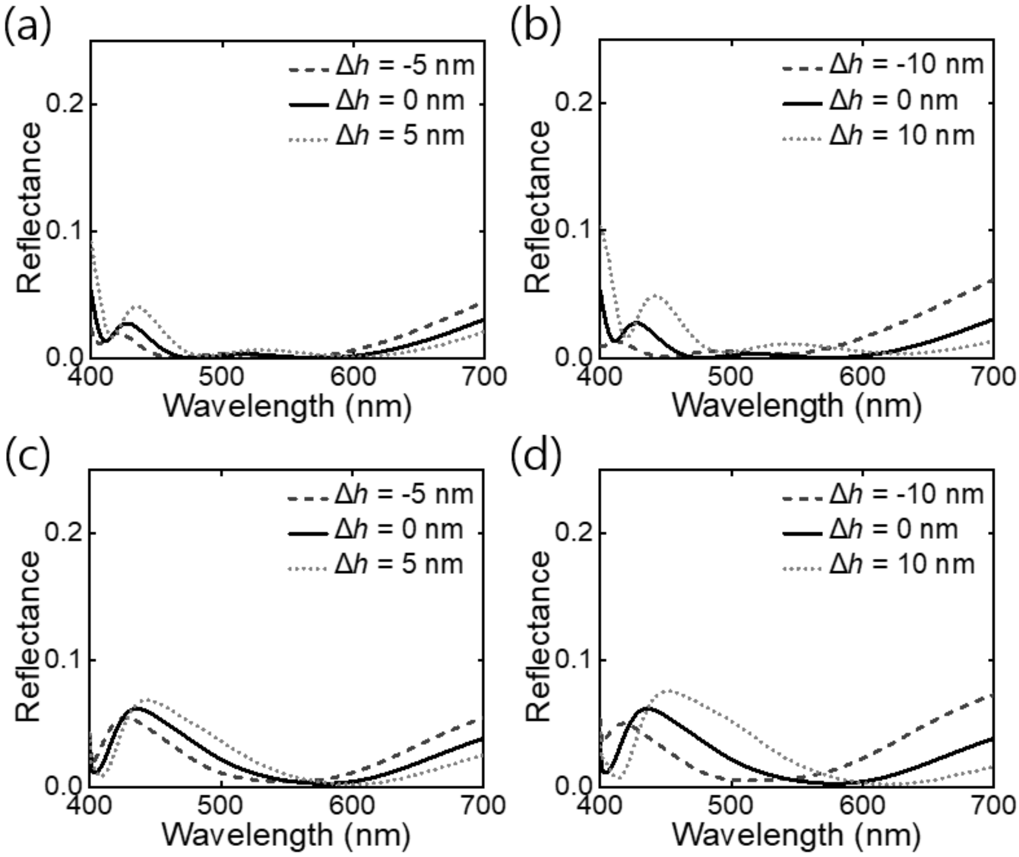

Figure S3. Effect of nanodisk height variations on reflectance, related to Figure 2. Fabrication tolerance analysis of optimized AAM designs at ϕ = 0° for θ = 0° (panels (a), (b)) and θ = 30° (panels (c), (d)). Starting from the optimized height (solid lines), the nanodisk height (h) was perturbed by ±5 nm (panels (a), (c)) and ±10 nm (panels (b), (d)), while all other parameters were kept unchanged. Dotted lines represent increased height and dashed lines represent decreased height.

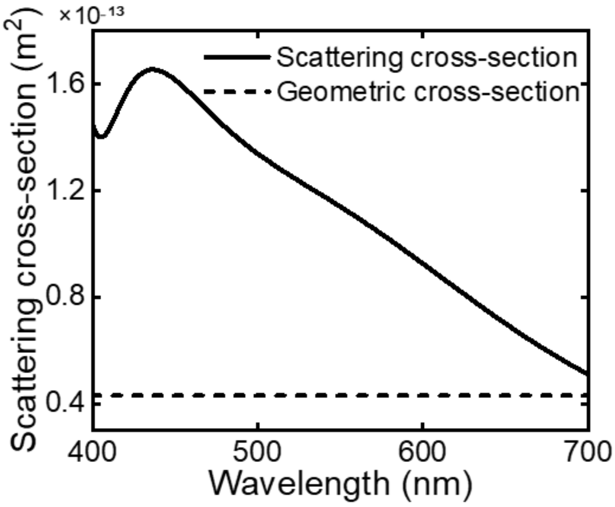

Figure S4. Scattering cross-section of an isolated TiO_2 nanodisk, related to Figure 3. Scattering cross-section (solid line) of a single TiO_2 nanodisk (r = 117 nm, h = 91 nm) on a Si substrate, calculated using a TFSF source with PML boundaries. The dashed line indicates the geometric cross-section ($\pi r^2 \approx 4.3 \times 10^{-14}$ m^2). A Mie resonance peak is observed at 436 nm with a magnitude approximately four times the geometric cross-section. No scattering feature is observed at longer wavelengths (500–700 nm).

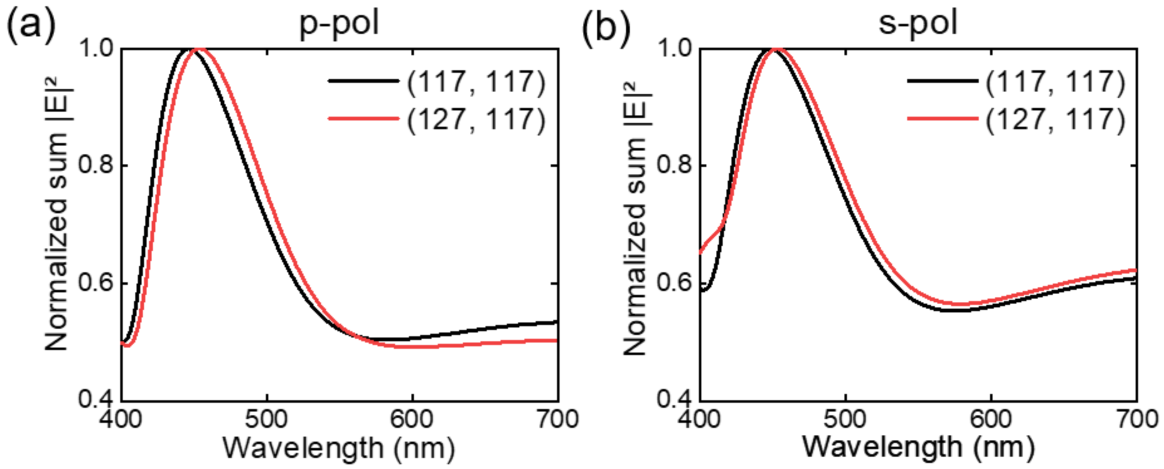

Figure S5. Near-field intensity spectra of the TiO_2 nanodisk array, related to Figure 3. Normalized incoherent sum of $|E|^2$ along the z-direction adjacent to the TiO_2 nanodisk for (a) p-polarization and (b) s-polarization at normal incidence. The line monitor is positioned at (x, y) = (137, 0) nm for p-polarization and (0, 137) nm for s-polarization, and remains fixed for all geometries. Black: circular nanodisk with major and minor radii (117, 117) nm; red: elliptical nanodisk with (127, 117) nm. In both polarizations, a Mie resonance peak is observed at 446–448 nm for the circular nanodisk, red-shifted by ~10 nm from the isolated-disk value (436 nm, Figure S4) due to inter-disk coupling. Upon major-axis elongation to 127 nm, the peak shifts

only slightly to 453–454 nm, indicating that the Mie resonance is largely insensitive to lateral geometry changes. No near-field enhancement is observed at longer wavelengths (500–700 nm).

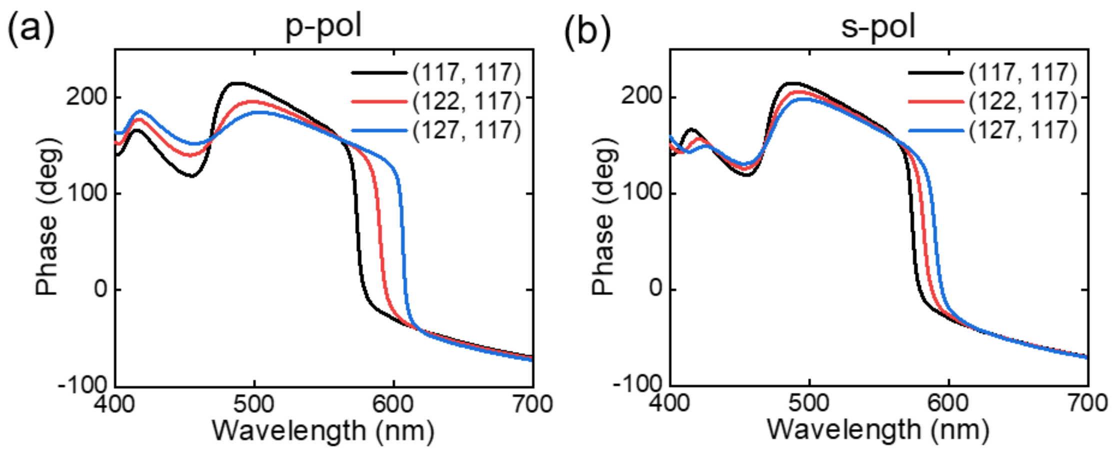

Figure S6. Reflection phase spectra showing polarization-dependent resonance splitting, related to Figure 3. Unwrapped reflection phase spectra with propagation phase removed at normal incidence: (a) p-polarization and (b) s-polarization. Three nanodisk geometries with major and minor radii of (117, 117), (122, 117), and (127, 117) nm are compared. For the circular nanodisk, a sharp phase transition is observed at 574 nm, which progressively red-shifts with increasing major-axis radius. The shift is larger for p-polarization (574 → 607 nm) than for s-polarization (574 → 591 nm), demonstrating polarization-dependent tuning enabled by the elliptical geometry. The broad feature at 400–500 nm shows negligible shift and polarization dependence.

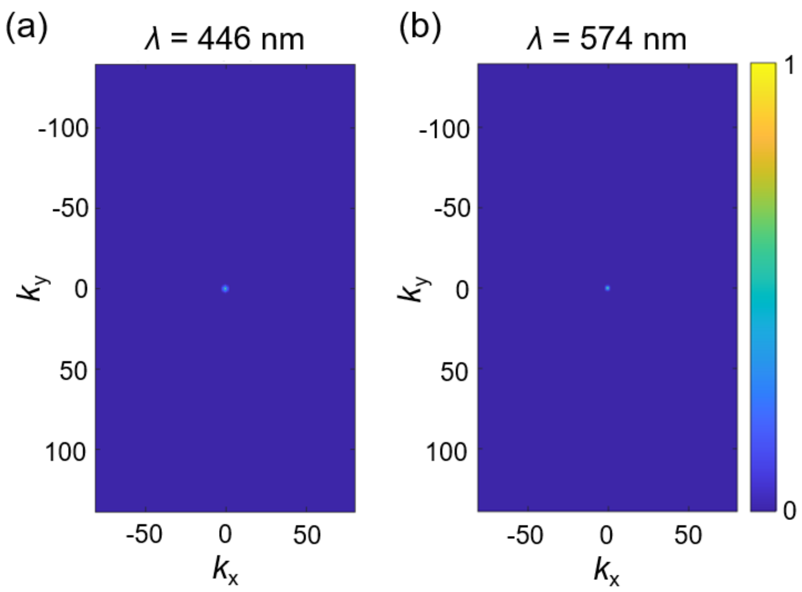

Figure S7. Spatial Fourier transform of the transmitted field, related to Figure 3. Normalized spatial Fourier transform of the transmitted electric field at a plane 50 nm below

the Si substrate at (a) 446 nm and (b) 574 nm for the circular nanodisk array with major and minor radii of (117, 117) nm at normal incidence, p-polarization. Only the zeroth diffraction order is present at both wavelengths, confirming the absence of higher-order diffraction and guided-mode resonance effects.

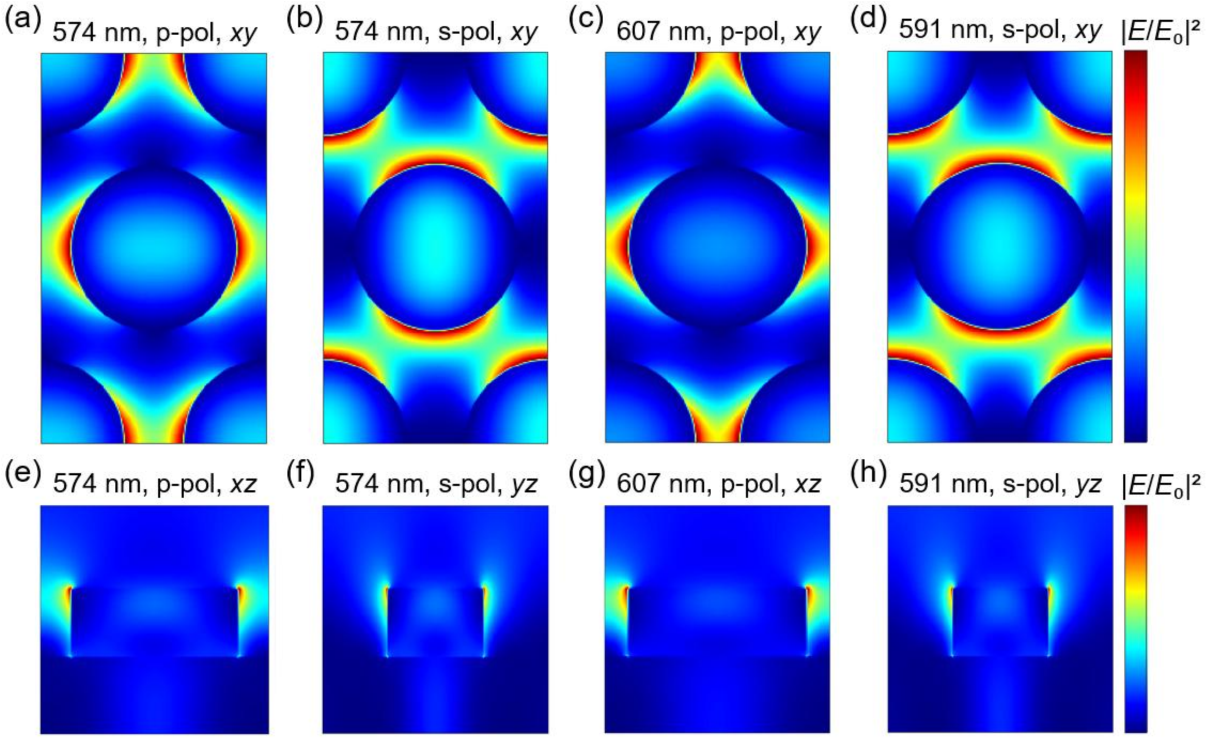

Figure S8. Total electric field intensity distributions at representative FP resonance wavelengths, related to Figure 3. $\left|\frac{E}{E_0}\right|^2$ distributions at normal incidence. Top row (a–d): *xy* cross-sections at the mid-height of the nanodisk. Bottom row (e–h): vertical cross-sections through the center of the nanodisk. (a) Circular nanodisk (117, 117) nm at 574 nm, p-polarization. (e) Same, *xz* cross-section at $y = 0$. (b) Same structure and wavelength, s-polarization. (f) Same, *yz* cross-section at $x = 0$. (c) Elliptical nanodisk (127, 117) nm at 607 nm, p-polarization. (g) Same, *xz* cross-section at $y = 0$. (d) Same structure at 591 nm, s-polarization. (h) Same, *yz* cross-section at $x = 0$. The far-field reflectance and phase are identical for both polarizations in the circular case (Figures 2b and S6), confirming mode degeneracy; the near-field difference between (a) and (b) arises from the directional asymmetry of the triangular lattice. The field distributions at the p-polarized (c, g, at 607 nm) and s-polarized (d, h, at 591 nm) cases are obtained at different resonance wavelengths, directly reflecting the polarization-dependent FP condition in the elliptical nanodisk geometry.

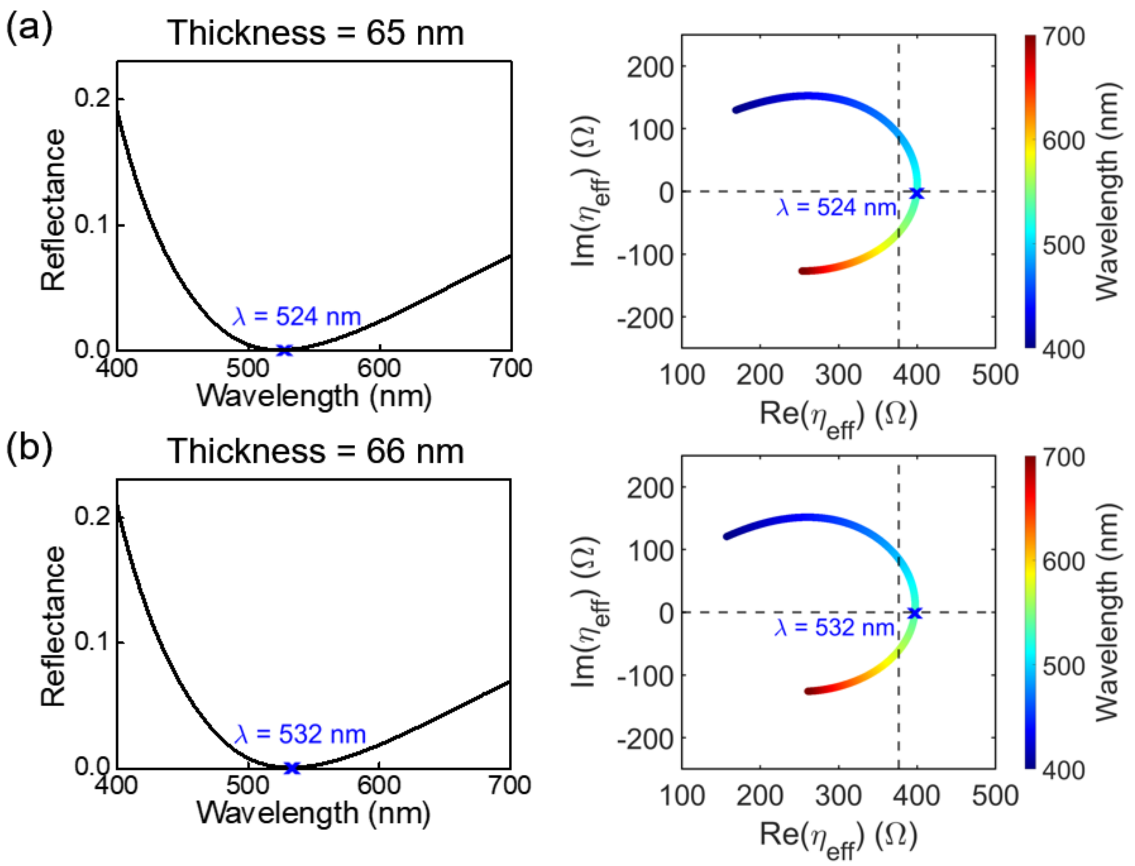

Figure S9. Impedance and reflectance analysis of Si_3N_4 single-layer anti-reflective coating (1-ARC), related to Figure 3.

Reflectance spectra (left) and effective transverse impedance trajectories (right) in the complex plane for a Si_3N_4 1-ARC on a silicon substrate at normal incidence. (a) A 65 nm-thick coating optimized to minimize the average reflectance across θ = 0°, 10°, 20°, 30°, and 40°. (b) A 66 nm-thick coating optimized specifically for θ = 30°, yielding minimum reflectance. The marked wavelengths correspond to reflectance minima and the impedance values closest to that of air.

Major, minor radii	f (Filling factor)	λ_{FP}, p-pol (sim)	λ_{FP}, s-pol (sim)	Splitting	λ_{FP}, p-pol (lamellar)	Difference, p-pol
(117, 117) nm	0.488	574 nm	574 nm	0 nm	676 nm	102 nm
(127, 117) nm	0.530	607 nm	591 nm	16 nm	706 nm	99 nm
(137, 117) nm	0.572	639 nm	607 nm	32 nm	728 nm	89 nm
(147, 117) nm	0.613	671 nm	622 nm	49 nm	746 nm	75 nm
(157, 117) nm	0.655	711 nm	636 nm	75 nm	768 nm	57 nm

Table S1. Fabry–Pérot resonance wavelengths for nanodisk arrays with increasing ellipticity, related to Figure 3. The filling fraction is calculated as $f = \frac{2\pi ab}{\sqrt{3}p^2}$ with p = 319 nm. Simulated FP wavelengths (λ_{FP}) are extracted from the maximum $\left|\frac{d\varphi}{d\lambda}\right|$ in the reflection phase spectra at normal incidence. The lamellar prediction for p-polarization is obtained from the

quarter-wave condition $\lambda_{\mathrm{FP}} = 4n_{\mathrm{eff}}h$ (h = 91 nm) using the arithmetic-mean effective dielectric constant, with self-consistent treatment of the TiO_2 dispersion. The corresponding harmonic-mean prediction for s-polarization is not shown, as the discrete nanodisk geometry deviates substantially from the 1D lamellar limit for this polarization. The difference between prediction and simulation decreases monotonically from 102 nm to 57 nm with increasing ellipticity, confirming convergence toward the lamellar limit.

	θ (°)	0	10	20	30	40
ϕ = 0°	Average *R* [AAM]	0.92%	0.90%	1.31%	2.28%	3.85%
	p (nm)	324	320	330	330	292
	h (nm)	94	92	92	92	86
	a (nm)	234	235	242	269	255
	b (nm)	234	232	238	224	202
ϕ = 15°	Average *R* [AAM]	-	0.93%	1.27%	2.45%	3.81%
	p (nm)	-	317	346	326	280
	h (nm)	-	91	97	91	88
	a (nm)	-	233	256	270	236
	b (nm)	-	232	238	228	195
ϕ = 30°	Average *R* [AAM]	-	0.90%	1.26%	2.48%	3.85%
	p (nm)	-	330	343	328	280
	h (nm)	-	91	93	91	83
	a (nm)	-	241	258	276	267
	b (nm)	-	239	242	227	182
	Average *R* [1-ARC]	3.93%	4.01%	4.16%	4.47%	4.92%
	t (nm)	64	64	64	66	67

Table S2. Optimized structural parameters and average reflectance of AAM and 1-ARC, related to Figure 2.

Results are shown for incidence angles (θ = 0°, 10°, 20°, 30°, and 40°) and azimuthal angles (ϕ = 0°, 15°, and 30°). Optimized AAM dimensions (*p*, *h*, *a*, *b*) and 1-ARC thickness (*t*) are listed, along with the corresponding reflectance averaged over p- and s-polarizations across the 400–700 nm range. Both structures were optimized using the same cost function.

	θ (°)	0	10	20	30	40
ϕ = 0°	Average *R* [AAM]	0.93%	0.93%	1.35%	2.39%	4.03%
	a (nm)	234	241	250	255	271
	b (nm)	234	230	228	228	214
ϕ = 15°	Average *R* [AAM]	-	0.93%	1.35%	2.46%	4.02%
	a (nm)	-	239	246	259	281
	b (nm)	-	230	228	223	212
ϕ = 30°	Average *R* [AAM]	-	0.93%	1.36%	2.50%	3.96%
	a (nm)	-	238	248	264	288
	b (nm)	-	230	224	222	215
	Average *R* [1-ARC]	3.92%	4.01%	4.16%	4.52%	5.10%
	Average *R* [2-ARC]	3.72%	3.79%	3.88%	4.17%	4.69%

Table S3. Re-optimized AAM parameters and comparison with 1-ARC and 2-ARC, related to Figure 4.

The nanodisk height (h = 91 nm) and unit cell period (p = 319 nm) were fixed by averaging the optimal values obtained across incidence angles (θ = 0°, 10°, 20°, 30°, and 40°) and azimuthal angles (ϕ = 0°, 15°, and 30°), and the lateral dimensions (a, b) were re-optimized for each angle combination. The 1-ARC thickness (65 nm) and 2-ARC thicknesses (Si_3N_4: 55 nm, SiO_2: 29 nm) were determined by minimizing the reflectance averaged over both polarizations, the 400–700 nm wavelength range, and all angle combinations.

	θ = 17.6°, ϕ = 17°	θ = 27.6°, ϕ = 17°	θ = 37.6°, ϕ = 17°
p-pol	2.27%	2.59%	3.25%
s-pol	1.43%	2.19%	4.19%
	θ = 17.6°, ϕ = 27°	θ = 27.6°, ϕ = 27° (origin)	θ = 37.6°, ϕ = 27°
p-pol	2.28%	2.69%	3.61%
s-pol	1.39%	2.06%	3.91%
	θ = 17.6°, ϕ = 37°	θ = 27.6°, ϕ = 37°	θ = 37.6°, ϕ = 37°
p-pol	2.25%	2.63%	3.48%
s-pol	1.45%	2.15%	4.03%

Table S4. Reflectance under angular deviations from the reference chief-ray angle, related to Figure 4.

Simulated reflectance values for p- and s-polarized light, averaged across the 400–700 nm wavelength range, at ±10° variations in both polar and azimuthal angles from the reference chief-ray angle (θ = 27.6°, ϕ = 27°).

Data S1. Resonance-mode identification, the polarization-dependent tuning mechanism, and field-distribution analysis of the aperiodic-anisotropic metasurface, related to Figure 2 and 3.

Identification of resonance modes

To identify the resonant modes supported by the TiO_2 nanodisk array, we performed a series of analyses at normal incidence on circular nanodisks (radius r = 117 nm). The scattering cross-section of an isolated TiO_2 nanodisk on a Si substrate, computed using a total-field/scattered-field (TFSF) source with perfectly matched layer (PML) boundaries, exhibits a peak at 436 nm with a magnitude approximately four times the geometric cross-section (Figure S4). This confirms a Mie resonance intrinsic to the individual nanodisk. Notably, no scattering feature is observed at longer wavelengths (500–700 nm).

The near-field intensity spectrum (incoherent sum of $|E|^2$ along the z-direction adjacent to the TiO_2 nanodisk in the periodic array) shows a peak at 446–448 nm for the circular nanodisk, red-shifted by ~10 nm from the isolated-disk value due to inter-disk coupling (Figure S5). Upon elongation of the major-axis radius to 127 nm, the peak shifts only slightly to 453–454 nm, indicating that the Mie resonance is largely insensitive to changes in lateral geometry. Notably, no near-field enhancement is observed at longer wavelengths (500–700 nm).

Mechanism of polarization-dependent tuning

We next examined the reflection phase spectra for nanodisks with major and minor radii of (117, 117), (122, 117), and (127, 117) nm at normal incidence (Figure S6). For the circular case, the p- and s-polarized phase spectra are identical, confirming degeneracy. As the major-axis radius increases, the sharp phase transition at longer wavelengths (574 nm for the circular nanodisk) progressively red-shifts, with a larger shift for p-polarization (574 → 607 nm) than for s-polarization (574 → 591 nm), yielding a splitting of 16 nm. In contrast, the broad feature at 400–500 nm shifts only slightly and shows negligible polarization dependence. This confirms that the polarization-dependent tuning originates mainly from the longer-wavelength resonance.

We attribute the sharp phase transition at longer wavelengths to a Fabry–Pérot (FP) resonance of the nanodisk array acting as an effective-medium slab. The polarization-dependent splitting can be explained by the anisotropic effective dielectric constant of the nanodisk array. To quantify this anisotropy, we consider the lamellar limit, where the elliptical nanodisks approach a line pattern. In this limit, the effective dielectric constants for the two polarizations are given by:

$$\varepsilon_{\parallel} = f\varepsilon_{\mathrm{TiO_2}} + (1-f)\varepsilon_{\mathrm{air}} \text{ (p} - \text{polarization, arithmetic mean)} \quad (1)$$

$$\frac{1}{\varepsilon_{\perp}} = \frac{f}{\varepsilon_{\mathrm{TiO_2}}} + \frac{(1-f)}{\varepsilon_{\mathrm{air}}} \text{ (s − polarization, harmonic mean)} \quad (2)$$

where $\varepsilon_{\mathrm{TiO_2}}$ and $\varepsilon_{\mathrm{air}}$ are the dielectric constants of anatase TiO_2 and air, respectively, and $f = \frac{2\pi ab}{\sqrt{3}p^2}$ is the filling fraction of the unit cell. Since the arithmetic mean always exceeds the harmonic mean, n_{eff}(p-pol) > n_{eff}(s-pol), and the FP quarter-wave condition $\lambda_{\mathrm{FP}} = 4n_{\mathrm{eff}}h$ is satisfied at a longer wavelength for p-polarization than for s-polarization. As the ellipticity increases, the difference between the two effective indices grows, leading to progressively larger splitting.

To verify this effective-medium interpretation, we performed additional simulations with progressively elongated nanodisks (major-axis radii of 137, 147, and 157 nm). As summarized in Table S1, the p–s splitting increases monotonically from 0 nm (circular) to 75 nm (most elongated). The lamellar model is not expected to be quantitatively accurate for finite elliptical nanodisks, as the discrete geometry deviates from the continuous 1D lamellar limit. Nevertheless, the difference between the lamellar prediction and the simulated p-polarized FP wavelength decreases monotonically with increasing ellipticity, confirming convergence toward the lamellar limit as the geometry approaches a line pattern. This trend demonstrates that the anisotropic effective index is the physical origin of the polarization-dependent FP splitting. We note that the lamellar approximation provides an upper bound for p-polarization, where the geometry increasingly resembles a continuous line; for s-polarization, the non-uniform width and discrete gaps of the elliptical disks deviate significantly from the continuous 1D lamellar limit, resulting in larger discrepancies between the FP wavelengths predicted by the lamellar harmonic mean and the actual simulations.

In addition to the FP resonance, the asymmetric phase feature observed at 400–500 nm (Figure S6) can be attributed to Fano-type interference between the weak Mie resonance and the broad FP background, consistent with the framework established for Si metasurfaces by Cordaro et al. [S4]. This interpretation is supported by the two asymmetric reflectance dips observed in the 400–500 nm range (Figure 2b). The Mie resonance in TiO_2 nanodisks is relatively weak owing to the lower refractive index and small height (h = 91 nm) compared to Si, as reflected in the modest scattering enhancement (~4× the geometric cross-section, Figure S4). As discussed above, the Mie resonance also shifts only modestly upon major-axis elongation, which explains why the Fano feature remains largely unchanged (Figure S6) and the polarization-dependent tuning is governed predominantly by the FP resonance. Overall, the Fano-mediated Mie contribution at shorter wavelengths and the FP resonance at longer wavelengths together account for the broadband anti-reflection performance across the visible spectrum. Separately,

spatial Fourier analysis of the transmitted field below the substrate confirmed that only the zeroth diffraction order is present, ruling out diffraction or guided-mode resonance effects (Figure S7).

Field distributions

We focus on the FP resonance region for the field analysis, as this is where the polarization-dependent splitting occurs upon major-axis elongation (Figure S6), whereas the broad feature at 400–500 nm remains largely unchanged. The total electric field intensity distributions ($\left|\frac{E}{E_0}\right|^2$) under normal incidence for both the circular (117, 117) and elliptical (127, 117) nm nanodisks under p- and s-polarized excitation are provided in Figure S8.

For the circular nanodisk at its FP resonance wavelength (574 nm), the far-field reflectance (Figure 2b) and phase (Figure S6) are identical for both polarizations, confirming mode degeneracy. Nevertheless, field distributions are shown for both p- and s-polarization, as the near-field patterns differ slightly due to the directional asymmetry of the triangular lattice, although this does not affect the far-field response. For the elliptical nanodisk (127, 117), field distributions are shown at the p-polarized FP resonance (607 nm; *xy* and *xz* cross-sections) and the s-polarized FP resonance (591 nm; *xy* and *yz* cross-sections), visually reflecting the polarization-dependent FP condition in the elliptical nanodisk geometry.

Field distributions at the Mie resonance wavelength (~446 nm) were also examined but are not shown, as the low refractive index and small height of the TiO_2 nanodisks result in weak field confinement that does not produce a distinctive modal pattern, consistent with the low-Q nature of the Mie resonance discussed above.

REFERENCES

S1. Jolivet, A., Labbé, C., Frilay, C., Debieu, O., Marie, P., Horcholle, B., Lemarié, F., Portier, X., Grygiel, C., Duprey, S., et al. (2023). Structural, optical, and electrical properties of TiO_2 thin films deposited by ALD: Impact of the substrate, the deposited thickness and the deposition temperature. Appl. Surf. Sci. *608*, 155214. 10.1016/j.apsusc.2022.155214.

S2. Philipp, H.R. (1973). Optical properties of silicon nitride. J. Electrochem. Soc. *120*, 295. 10.1149/1.2403440.

S3. Palik, E.D. (1998). Handbook of Optical Constants of Solids (Academic Press).

S4. Cordaro, A., van de Groep, J., Raza, S., Pecora, E.F., Priolo, F., and Brongersma, M.L. (2019). Antireflection high-index metasurfaces combining Mie and Fabry-Pérot resonances. ACS Photonics *6*, 453–459. 10.1021/acsphotonics.8b01406.
